# The Knockdown of ACTL6A Enhances the Radiosensitivity of Esophageal Squamous Cell Carcinoma by Modulating the Wnt/β‐Catenin Signaling Pathway

**DOI:** 10.1096/fj.202501681R

**Published:** 2025-10-25

**Authors:** Shuo Zhou, Tongyou Sun, Likun Liu, Dong Guo, Xueyuan Zhang, Wenbin Shen, Shaolin Gao, Shuchai Zhu

**Affiliations:** ^1^ Department of Radiation Oncology The Fourth Hospital of Hebei Medical University Shijiazhuang, Hebei China; ^2^ Department of Radiation Oncology Chengde Central Hospital Chengde, Hebei China; ^3^ Department of Radiation Oncology Weifang People's Hospital Weifang, Shandong China; ^4^ Department of Thoracic Surgery The Second Hospital of Hebei Medical University Shijiazhuang, Hebei China

**Keywords:** ACTL6A, epithelial‐mesenchymal transition, esophageal squamous cell carcinoma, radiosensitivity, Wnt/β‐catenin signaling pathway

## Abstract

Actin‐like protein 6A (ACTL6A) is thought to be associated with the survival and prognosis of patients with a variety of human cancers. This study investigates the effect of ACTL6A knockdown on ESCC radiosensitivity and explores molecular mechanisms that may enhance radiotherapy efficacy. The ACTL6A expression level was increased in esophageal squamous carcinoma cells after radiation irradiation. The protein expression level of ACTL6A in tumor tissue samples of clinical esophageal squamous cell carcinoma patients was analyzed by immunohistochemistry, and it was found that the prognosis of the high expression group was worse than that of the low expression group. Further knocking down the ACTL6A gene in esophageal squamous cell carcinoma cells, it was found that ACTL6A could regulate the proliferation, migration, invasion, DNA damage repair, cell cycle, and apoptosis of esophageal squamous cell carcinoma cells, which further affected the radiosensitivity of esophageal squamous cell carcinoma cells. Through functional enrichment analysis of gene set enrichment and validation of the mechanism using the Wnt pathway inhibitor XAV939, it was shown that ACTL6A is involved in the regulation of the Wnt/β‐catenin signaling pathway. Knockdown of ACTL6A can inhibit the activity of this pathway, thereby increasing the radiosensitivity of esophageal squamous cell carcinoma. ACTL6A may become an important therapeutic target for esophageal squamous cell carcinoma, providing a necessary theoretical basis for future treatment strategies.

## Graphical abstract

ACTL6A, A new radiosensitizing target for esophageal squamous cell carcinoma?

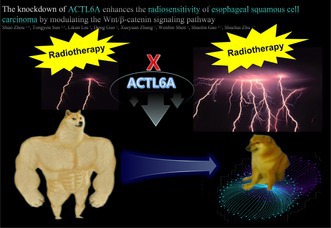

**Abbreviations**
ACTL6A: actin‐like protein 6A
ANOVA: Analysis of Variance
BAF/PBAF: BRG1/BRM‐associated factor/Polybromo‐associated BAF complex
CCK‐8: Cell Counting Kit‐8
CDC25C: cell division cycle 25C
CDK1: cyclin‐dependent kinase 1
cDNA: complementary DNA
DFS: disease‐free survival
DSB: DNA double‐strand break
EMT: epithelial‐mesenchymal transition
ESCC: esophageal squamous cell carcinoma
FBS: fetal bovine serum
FCM: flow cytometry
GAPDH: glyceraldehyde‐3‐phosphate dehydrogenase
GSEA: Gene Set Enrichment Analysis
HEEC: human esophageal epithelial cells
IHC: immunohistochemistry
NC: negative control
OS: overall survival
PI: propidium iodide
PVDF: polyvinylidene fluoride
qRT‐PCR: quantitative real‐time polymerase chain reaction
RT: radiotherapy
SDS‐PAGE: sodium dodecyl sulfate‐polyacrylamide gel electrophoresis
shRNA: short hairpin RNA
SPF: specific pathogen‐free
SWI/SNF: Switch/Sucrose non‐fermentable
TNM: Tumor, node, metastasis
Wnt/β‐catenin: Wingless/Integrated‐beta‐catenin
γH2AX: phosphorylated histone H2AX

## 1 Introduction

Esophageal cancer is the sixth leading cause of cancer‐related death globally. It is a highly aggressive disease with significant incidence and mortality rates [1]. After diagnosis, the predicted 5‐year survival rate is between 10% and 30%, indicating that esophageal cancer has a poor prognosis [2]. Esophageal squamous cell carcinoma (ESCC) and esophageal adenocarcinoma are the two predominant types of this cancer, with ESCC being more common [3, 4]. Radiotherapy is one of the primary treatment options for ESCC, especially in locally advanced cases, and is utilized as adjuvant therapy after surgery [5]. The efficacy of radiotherapy significantly differs among patients, primarily due to tumor cell radiosensitivity variations. The resistance of ESCC cells to radiation reduces treatment efficacy, resulting in tumor recurrence and distant metastasis [6]. Therefore, enhancing the radiosensitivity of ESCC is crucial for improving treatment efficacy and patient survival rates.

Actin‐like protein 6A (ACTL6A) is a cytoskeletal protein homologous to β‐actin and is an essential component of the SWI/SNF chromatin remodeling complex. The complex regulates the transcriptional activation and suppression of genes through ATP‐dependent chromatin remodeling [7]. ACTL6A regulates cell proliferation, differentiation, and stemness maintenance through interactions with other BAF/PBAF complex components, including BRG1 and INI1 [8]. It primarily facilitates the equilibrium of epidermal precursor cell growth and differentiation in healthy tissues, while its function is frequently disrupted in the tumor environment, thus promoting cancer cell metastasis.

Several cancers have aberrantly high ACTL6A expression, which is directly associated with the onset, metastasis, and therapeutic resistance of malignant tumors [9]. In cancers, including lung and breast cancers, ACTL6A enhances the progression, metastasis, and invasion abilities of tumor cells and is associated with a poor prognosis [10]. ACTL6A Overexpression in colon cancer enhanced colon cancer cell motility and invasion and may act as an epithelial‐mesenchymal transition (EMT) activator in vitro [11]. EMT is distinguished by heightened levels of vimentin, which serves as a migratory marker, and diminished expression of the epithelial marker E‐cadherin [12]. In ovarian cancer, ACTL6A regulates stem cell‐related gene expression by modulating chromatin remodeling, thereby preserving the stem‐like characteristics of tumor cells [13]. A previous study reported that ACTL6A is linked to immune infiltration in cancer [14]. The specific roles of ACTL6A in its association with radiation sensitivity are unclear. Therefore, this study aimed to investigate the role of ACTL6A on the radiosensitivity of esophageal cancer cells and the underlying mechanism to enhance the efficacy of radiotherapy for patients with cancer and address the treatment limitation posed by tumor tolerance.

## 2 Materials and Methods

### 2.1 Analysis of Clinical Data

With the approval of the review committee of the Fourth Hospital of Hebei Medical University (Approval No. 2024 KY112), endoscopic biopsy tissue samples and clinical data of 30 patients with esophageal squamous cell carcinoma confirmed by pathology and treated with radiotherapy and chemotherapy at the Fourth Hospital of Hebei Medical University in 2016 and 2022 were collected for a retrospective study. As this was a retrospective study, the requirement for informed consent was waived. The primary outcome of this study was disease‐free survival (DFS). Patients were followed up from recurrence, death, or the last monitoring, with recurrence as the endpoint. The primary outcome was overall survival (OS). Patients were followed up from the date of surgery until death due to esophageal cancer or the last follow‐up. The data cut‐off date was September 2024. Both DFS and OS were measured in months.

The expression characteristics of ACTL6A protein and its impact on the survival prognosis of patients undergoing radiotherapy and chemotherapy were explored through immunohistochemical experiments.

Interpretation of immunohistochemical results: Under the microscope, ACTL6A was mainly located in the cytoplasm and nucleus. The immunohistochemical results were independently and blindly evaluated by two chief pathologists. The results were judged under an optical microscope, following the assessment method of Hou et al. [15]. Each slide was observed under a microscope to cover the entire field of view containing tumor tissue, and the staining intensity and proportion of positive cells were evaluated. Staining intensity assessment: 0 points (no staining), 1 point (light yellow), 2 points (brownish yellow), 3 points (brown). Proportion of positive cells: 0 points (0%), 1 point (< 10%), 2 points (10%–50%), 3 points (51%–80%), 4 points (> 80%). Tumor tissue specimens with a total score greater than 4 were defined as high expression of ACTL6A, and those with a score of 4 or less were defined as low expression.

### 2.2 Bioinformatic Analysis

The gene expression omnibus dataset (GSE53624) was used to analyze the gene expression profiles of ESCC tissues, which include 179 ESCC samples and 179 adjacent non‐tumor tissues. The filter criteria were set as |LogFC| ≥ 1 and adjusted *p* < 0.05. The “Survival Miner” R package was employed to generate survival curves using the Kaplan–Meier method. Subsequently, the expression data from patients with ESCC were used to enrich and examine the key signaling pathways regulated by ACTL6A.

### 2.3 Cell Lines and Irradiation

Human esophageal epithelial cells (HEEC) and various ESCC cell lines (KYSE450, TE1, KYSE520, KYSE150, KYSE70) were employed in this study, sourced from the Tumor Research Institute at the Fourth Hospital of Hebei Medical University, China. The cells were cultured in RPMI 1640 medium enriched with 10% fetal bovine serum (FBS) and 1% penicillin–streptomycin. They were incubated at 37°C with 5% CO_2_. A 6 MV monoenergy Siemens linear accelerator (Siemens, Buffalo Grove, IL, USA) was used to irradiate the ESCC cells with X‐rays at 3 Gy/min. Cells were harvested at specific intervals after setting the source‐skin distance at 100 cm for subsequent experimental analysis.

### 2.4 Plasmids and Lentiviral Construction

The full‐length complementary DNA (cDNA) of human ACTL6A was cloned, and the ACTL6A knockout esophageal cancer cell lines were created using adenoviruses that expressed the mCherry‐GFP‐LC3 fusion protein lentivirus and ACTL‐6AshRNA (Genecon, Shanghai, China). The shRNA adenovirus was introduced into adherent ESCC cells according to the manufacturer's instructions. Puromycin was administered at 2 μg/L for selection, resulting in the establishment of stable ESCC cell lines.

### 2.5 Quantitative Real‐Time Polymerase Chain Reaction (qRT‐PCR)

As directed by the manufacturer, total RNA was extracted using the TRIzol reagent (Invitrogen, Carlsbad, CA, USA). The ACTL6A knockdown cells and control cells were taken for full contact and reaction with TRIzol, respectively. A NanoDrop ND‐1000 Spectrophotometer (Thermo Fisher Scientific, Waltham, MA, USA) was used to quantify the amount of RNA in the cells, which typically had an A260/A280 ratio above 2.0. The 1% agarose gel electrophoresis was used to evaluate the integrity and quality of the RNA. Using the TransScript R One‐Step gDNA Removal and cDNA Synthesis SuperMix (TransGen Biotech Co. Ltd., Beijing, China) instructions, 2.0 μg of each extracted RNA was added in a 20.0 μL volume of the reaction with the primer. The reverse transcription was then carried out under the PCR conditions of 42°C for 30 min and 85°C for 5 s. 10 μL of 2 × TransStart Top Green qPCR SuperMix, 1.0 μL of cDNA template, 0.4 μL of each forward and reverse gene‐specific primer, 0.4 μL of passive reference DyeII, and 7.8 μL of RNase‐free H2O were used in the 20.0 μL volume used for the real‐time PCR tests. The PCR was run at 94°C for 30 s, then for 40 cycles of 94°C for 5 s, 60°C for 30 s, 64°C for 5 s, and finally 95°C for 35 s. Every sample was evaluated three times. This reference gene was GAPDH. A 2^−∆∆T^ technique was used to determine the relative expressions of the ACTL6A [16]. qPCR was performed in triplicate on each of the three separate samples. GraphPad Prism 9.0.5 software (GraphPad Software Inc., La Jolla, CA, USA) was used to do statistical analysis. Tukey's post hoc test for multiple comparisons (*p* < 0.05) was used after one‐way ANOVA. The following primers were used:

ACTL6A: 5′‐TCAGAGGCACCGTGGAATACTAG‐3′ (forward), 5′‐AGGACATAGCCATCGTGGACTG‐3′ (reverse); and GAPDH: 5′‐GCTGAACGGGAAGCTCACTG‐3′ (forward), 5′‐GT GCTCAGTGTAGCCCAGGA‐3′ (reverse).

### 2.6 Cell Counting Kit‐8 Assay

The KYSE150 and KYSE70 cells in the control and ACTL6A knockdown groups were diluted to a 2 × 10^4^ cells/mL concentration. Subsequently, 100 μL of cell suspension was added to each well of a 96‐well plate, followed by radiotherapy after cell adhesion. At the specified time intervals after irradiation (24, 48, 72, and 96 h), 10 μL of CCK‐8 reagent (MedChemExpress, Princeton, New Jersey, USA) was added to each well. After incubating for 2 h, a microplate reader (wavelength 450 nm) was used to measure the absorbance.

### 2.7 Colony Formation Assay

Control and ACTL6A knockdown cells of KYSE150 and KYSE70 cell lines were cultured in six‐well plates and irradiated with different doses of radiation. The cells were cultured in a medium for 14 days, and subsequently fixed and stained. Subsequently, the colony was counted by considering cell clusters with > 50 cells as one colony. GraphPad Prism software (version 9.5; GraphPad Software Inc., La Jolla, CA) was used to generate the survival curve (SF). The single‐hit multi‐target model, expressed by the formula [SF = 1 − (1‐e‐D/D0) N], was used to generate the dose–response curve.

### 2.8 Western Blotting

Western blotting was used to assess the protein expression, and GAPDH was utilized as an internal control for quantification. Total proteins underwent separation using SDS‐PAGE before being transferred to a polyvinylidene fluoride (PVDF) membrane. Blocking of the PVDF membranes was carried out using 5% skim milk for 30 mins. Subsequently, the PVDF membranes underwent incubation overnight with primary antibodies at 4°C. On the subsequent day, the PVDF membrane's unbound primary antibody was exposed to a secondary antibody for 1.5 h. An appropriate volume of ECL‐A and ECL‐B (New Cell Science Co. Ltd.) was mixed in a 1:1 ratio to formulate the chemiluminescent reagent (freshly prepared and shielded from light). The reagent was administered to the PVDF membrane and incubated for 1 min before placing the membrane in a chemiluminescence imager for detection. Each Western blotting analysis was performed thrice. In this study, the concentration of the acrylamide gel was determined based on the molecular weight of the target protein: for proteins with a molecular weight greater than 100 kDa, an 8% acrylamide gel was used; for proteins with a molecular weight between 50 and 100 kDa, a 10% acrylamide gel was used; for proteins with a molecular weight less than 50 kDa, a 12% acrylamide gel was used. The total protein loading in each lane was 30 μg, which was determined through pre‐experiments and was optimized to ensure moderate band signal intensity (avoiding saturation) and to form a stable and comparable detection window between different samples, meeting the requirements of quantitative analysis.

### 2.9 Cell Apoptosis Analysis

Cell apoptosis was assessed in KYSE150 and KYSE70 cells. The control and shACTL6A cells, 1 × 10^6^, were cultured in six‐well plates and exposed to a single dose of 6 Gy radiation after cell attachment. Cells were harvested by centrifugation 24 h after radiation and washed with pre‐chilled PBS. Annexin V‐FITC and propidium iodide (PI) (Biolgend Inc., San Diego, CA, USA) were added to each centrifuge tube and incubated at room temperature for 5 min. A flow cytometer (Beckman Coulter) was used to analyze the fluorescence of Annexin V‐FITC.

### 2.10 Cell Cycle Assay

A six‐well plate was inoculated with ESCC cells at 3 × 10^5^ per well and subjected to a 6 Gy dose of X‐ray radiation. Cells were trypsinized after 48 h. After centrifugation, the cells were stained with DNase‐free RNase A solution (Sigma‐Aldrich, 69 182) and PI. FlowJo cytometry software (version 10) served to analyze the distribution of cells across the various phases of the cell cycle.

### 2.11 Cell Matrigel Invasion Assay

Matrigel‐coated Transwell inserts (Corning Inc., New York, USA) were inoculated with 1 × 10^5^ cells suspended in 200 μL of RPMI‐1640 media supplemented with 5% FBS (Certified Fetal Bovine Serum, C04001‐050X10). Additionally, 600 μL of RPMI‐1640 media supplemented with 20% FBS was introduced into the lower compartment to establish a chemoattractant gradient conducive to cell migration. Following a 24 h incubation period, any leftover cells on the insert's top surface were meticulously cleared with a cotton swab. Once the cells had migrated beneath the membrane, they were fixed with 4% paraformaldehyde for 20 min and stained with 0.1% violet crystal for 15 min. A Nikon Ti2 fluorescent microscope was used to visualize the migrated cells, and the number of labeled cells was counted and examined.

### 2.12 Cell Immunofluorescence Staining

ESCC cells were inoculated onto glass slides and cultured overnight, and the slides were washed twice with PBS, with a 5‐min interval between washes. After 4% paraformaldehyde fixation for 20 min, the cells were permeabilized using 0.5% Triton X‐100 for an additional 20 min. Subsequently, 10% goat serum was applied to the slides for 30 min to prevent nonspecific binding. The cells were incubated overnight at 4°C after adding primary antibodies. After transfection, γ‐H2AX expression was identified using the red fluorescent secondary antibody. Protein localization was observed through DAPI staining of the nuclei. DAPI was used to stain the nuclei, which were then visualized through confocal microscopy (Nikon A1, Japan).

### 2.13 Xenograft Subcutaneous Tumor Experiment

According to previous related studies [17]. We used 20 female/c nude mice aged 5 weeks (Huafukang Biotechnology Co. Ltd., Beijing, China). First, input the animal number into the spreadsheet software (such as Excel), and then use the random function to generate a random number. For example, in Excel, use the RAND function to generate a random number for each animal, then sort the animals according to the size of the random number, and finally group them according to the sorting result. Five mice were randomly assigned to each group (There were four groups: control group, ACTL6A knockdown group, radiotherapy alone group and radiotherapy + ACTL6A knockdown group). Individual ventilation cages under specified pathogen‐free conditions were utilized to house the mice. The KYSE150 control cells (1 × 10^7^) or ACTL6A knockdown cells (1 × 10^7^) were resuspended in 500 μL of serum‐free medium and then injected into female mice. Local irradiation (5 Gy in three fractions) was administered under isoflurane inhalation anesthesia on the 8th day after donation [16]. The length (a) and breadth (b) of the tumor were measured every 4 days using the formula *V* = ab2/2 to calculate the tumor volume. After 24 days, the mice were anesthetized by inhaling isoflurane and euthanized through cervical dislocation, after which the tumors were removed and weighed. All animal handling and experimental procedures adhered to the protocols established by the Animal Care and Use Committee at the Fourth Hospital of Hebei Medical University (SYXK [Hebei Province] 2022‐011).

### 2.14 Immunohistochemistry

Immunohistochemistry was used to confirm the target protein expression. The samples were first fixed in 4% formaldehyde, followed by dehydration using a graded ethanol series, clearing with xylene, and finally embedding in paraffin. Three sections were prepared for each target protein from each paraffin block using serial 4‐μm slicing. After being dewaxed in xylene and dried at 60°C, the sections were rehydrated using distilled water. Following antigen retrieval, endogenous peroxidase activity was inhibited. The sections were treated with goat serum to reduce nonspecific binding. The primary antibodies were diluted 1:100 and incubated overnight at 4°C. Sections were incubated with the enhancement solution for 30 min at 37°C to facilitate fluorescence signal amplification. The slices were incubated at 37°C for 40 min with an enzyme‐labeled goat anti‐rabbit immune globulin G polymer. Hematoxylin counterstaining was performed after 3,3′‐diaminobenzidine staining. Coverslips were used to secure the components. Staining intensity was evaluated microscopically. Intense positive staining manifested as brownish, moderate positive as brownish‐yellow, and weak positive as light yellow.

### 2.15 Statistical Analysis

For the analysis of data, R software (version 3.5.2) and GraphPad Prism (version 9.5; GraphPad Software Inc., CA, USA) were utilized. The outcomes from three distinct experiments are expressed as mean ± standard deviation. Comparisons among several groups were made using a one‐way analysis of variance, and differences between two groups were examined with a two‐tailed Student's *t*‐test. A *p*‐value below 0.05 was deemed statistically significant.

## 3 Results

### 3.1 The Relationship of ACTL6A Expression Level and Patient Prognosis in ESCC

**FIGURE 1:**
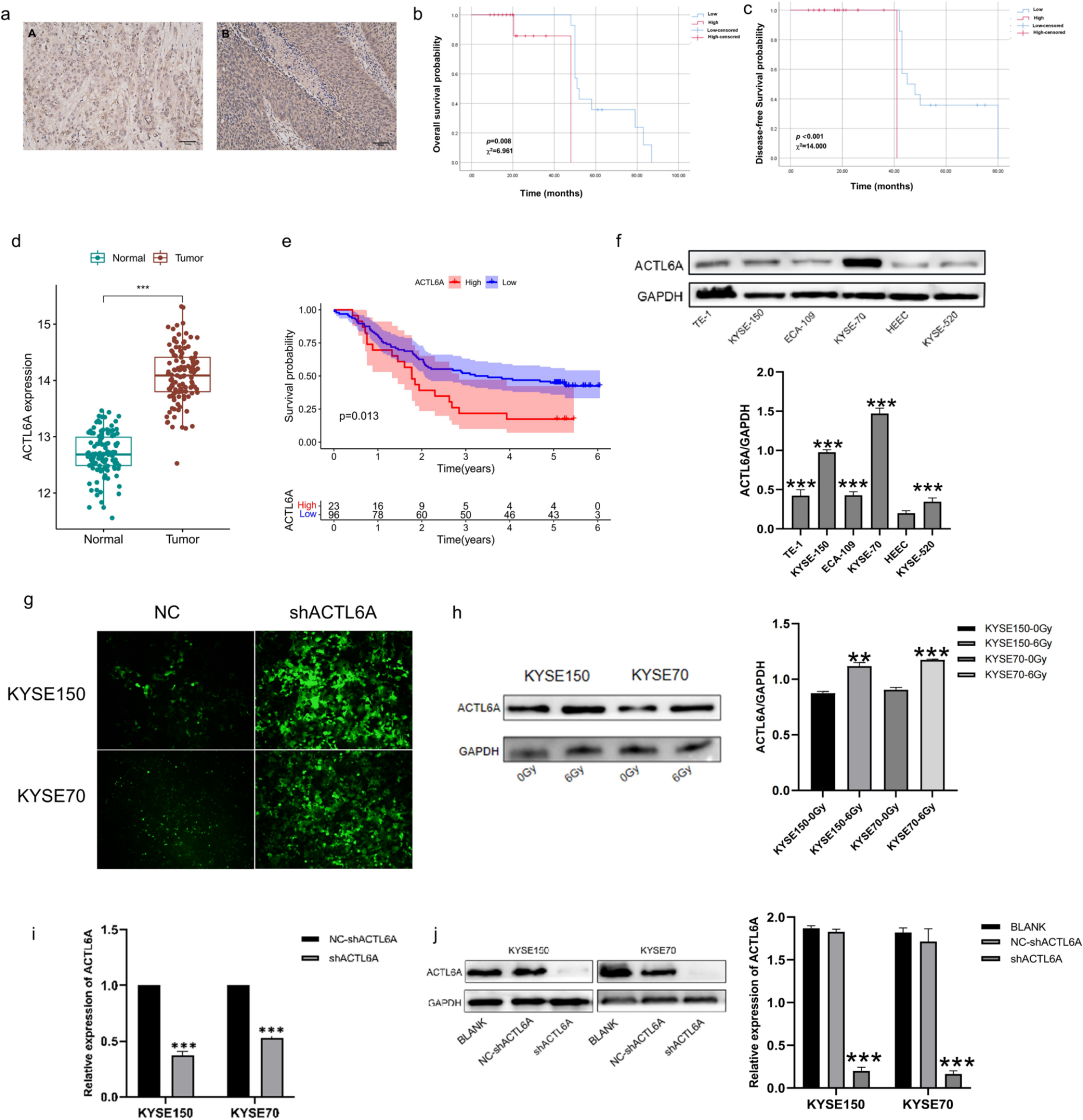
Relationship between the expression of ACTL6A and prognosis in ESCC and the construction of ACTL6A knockdown stable cell line. (a) Expression of ACTL6A protein in ESCC tissues (High expression of ACTL6A protein in ESCC tissues (A) and Low expression of ACTL6A protein in ESCC tissues (B)). (b, c) OS (b) and DFS (c) survival curves for the whole data set (patients were divided into two groups according to ACTL6A expression level in patient tumor tissue). (d) ACTL6A expression is higher in ESCC tissues compared to normal tissues. (e) Kaplan–Meier analysis of overall survival (OS) related to ACTL6A expression in ESCC patients. (f) Expression of ACTL6A in ESCC cell lines and HEEC cells. (g) Validation of ACTL6A knockdown efficiency using fluorescence microscopy. (h) Effect of radiation irradiation on ACTL6A expression levels in esophageal squamous carcinoma cells. (i) Validation of ACTL6A knockdown efficiency using qRT‐PCR. (j) Validation of ACTL6A knockdown efficiency using Western blot analysis. *p < 0.05, **p < 0.01, ***p < 0.001.

We further explored the expression characteristics of ACTL6A protein and its impact on the survival prognosis of patients with esophageal squamous cell carcinoma who received radiotherapy and chemotherapy by using immunohistochemical experiments in 30 tissue specimens. The immunohistochemical results showed that the expression of ACTL6A protein was mainly in the cytoplasm and nucleus (Figure 1a), among which 14 cases (46.7%) were low expression (A) and 16 cases (53.3%) were high expression (B). Combining the clinical pathological data of each group of patients, it was found that the expression of ACTL6A protein was closely related to T stage, N stage, and pathological stage (*p* < 0.05), as shown in Table 1. The survival analysis results showed that the overall survival of patients with low expression of ACTL6A protein was significantly better than that of the high expression group, and the difference was statistically significant (*p* = 0.008). Further DFS analysis showed that the overall survival of the low expression group of ACTL6A protein was significantly better than that of the high expression group, and the difference was statistically significant (*p* < 0.001), as shown in Figure 1b,c.

**TABLE 1:** Relationship between ACTL6A protein expression levels and clinicopathological features in tissue samples of esophageal squamous cell carcinoma patients.

Variables	Expression of ACTL6A		Exp(B)	*p*
	Low, *n* (%)	High, *n* (%)		
**Sex**			2.407	0.301
Male	9 (30.0)	13 (43.3)		
Female	5 (16.7)	3 (10.0)		
**Age**			2.584	0.642
≤ 60	6 (20.0)	7 (23.3)		
> 60	8 (26.7)	9 (30.0)		
**Smoking history**			2.314	0.264
Never	9 (30.0)	7 (23.3)		
Ever	5 (16.7)	9 (30.0)		
**Drinking history**			0.800	0.765
Never	8 (26.7)	10 (33.3)		
Ever	6 (20.0)	6 (20.0)		
Tumor location			0.933	0.946
Neck/upper	4 (13.3)	5 (16.7)		
Middle/lower	10 (33.3)	11 (36.7)		
**T stage**			8.883	0.003
T0–1	12 (40.0)	4 (13.3)		
T2–3	2 (6.7)	12 (40.0)		
**N stage**			8.036	0.013
N0–1	12 (40.0)	10 (33.3)		
N2–3	2 (6.7)	6 (20.0)		
**Pathological stage**			12.660	0.001
I–II	11 (36.7)	4 (13.3)		
III	3 (10.0)	12 (40.0)		

### 3.2 Molecular Characteristics of ACTL6A in Transcriptome Cohorts

Analysis of data from the GSE53625 cohort showed that ACTL6A expression levels were upregulated in ESCC tissues compared to normal tissues (Figure 1d). Survival analysis showed that in the GSE53625 cohort, patients with high ACTL6A expression had worse clinical outcomes than those with low ACTL6A expression (*p* = 0.013, Figure 1e). These results indicated that ACTL6A expression was altered between ESCC tissues and normal tissues and speculated an important regulatory mechanism of ACTL6A in ESCC tumorigenesis. The ACTL6A protein levels were confirmed using Western blotting in HEEC and several ESCC cell lines (ECA109, TE1, KYSE70, KYSE150, and KYSE520). Higher levels of ACTL6A were observed in ESCC cell lines than in HEEC (*p* < 0.05), with the KYSE150 and KYSE70 cell lines exhibiting the highest expression (Figure 1f). The ACTL6A expression level in esophageal squamous carcinoma cells (Figure 1h), which were utilized for ACTL6A knockdown. Fluorescence microscopy revealed successful transfection of the virus into ESCC cells (Figure 1g); both qRT‐PCR (Figure 1i) and Western blotting (Figure 1j) revealed that ACTL6A expression was significantly decreased (*p* < 0.001), confirming effective ACTL6A knocked down.

### 3.3 ACTL6A Knockdown Inhibits Proliferation and Radiation Resistance in ESCC Cells

**FIGURE 2:**
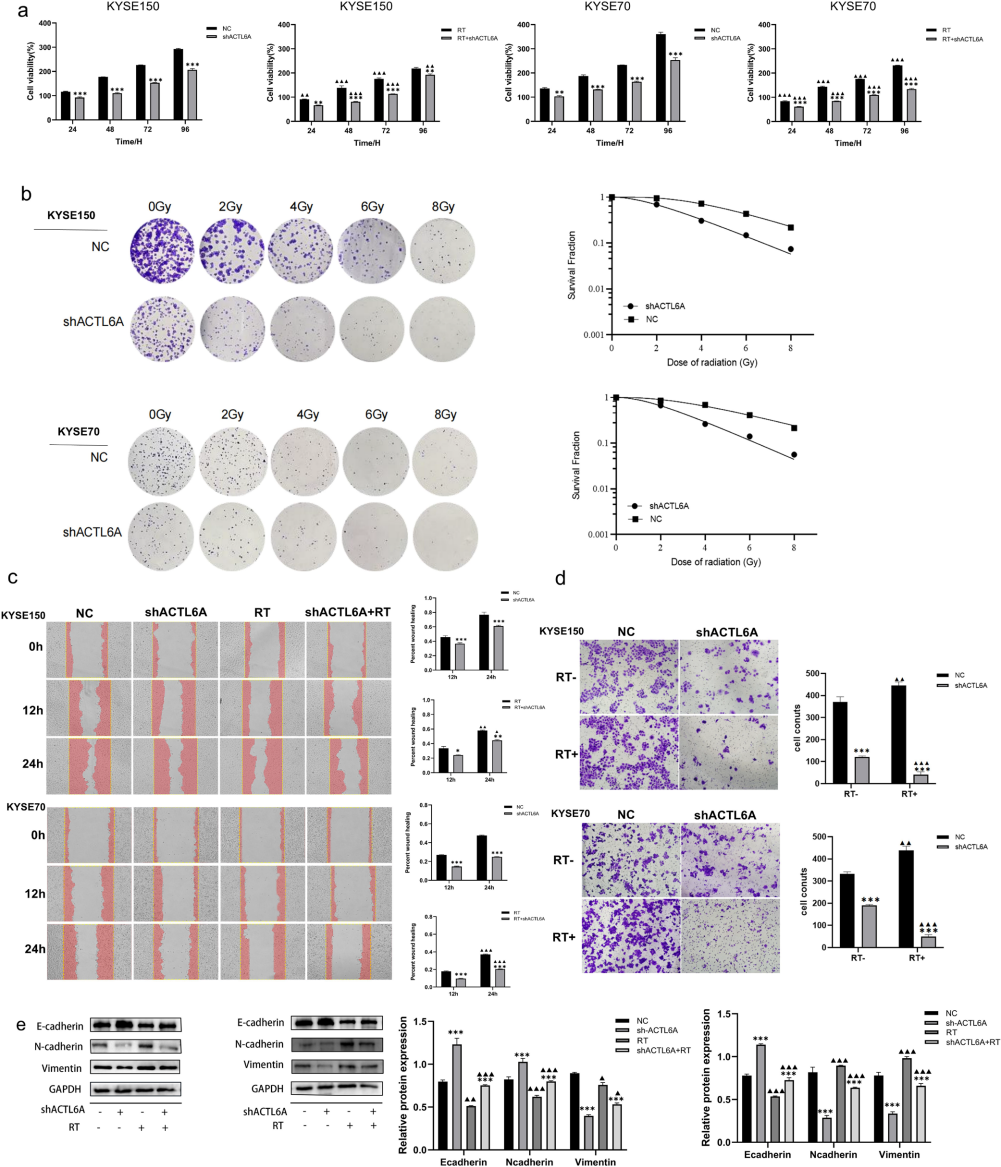
The promotion of ACTL6A on the proliferation, invasion, migration, and radiation resistance of ESCC cells. (a) Cell proliferation was assessed using the CCK‐8 assay. (b) The radiosensitivity of KYSE150 and KYSE70 cells was determined using the colony formation assay. (c) The migration capability of ESCC cells was evaluated using the wound healing assay. (d) The invasion ability of ESCC cells was studied using the TransWell assay (magnification ×200). (e) Western blot was used to detect the expression levels of EMT‐related proteins. Comparisons with the NC group are indicated by asterisks *p < 0.05, **p < 0.01, and ***p < 0.001; comparisons with the corresponding nonirradiated group are indicated by triangles ^▲^ *p* < 0.05, ^▲▲^ *p* < 0.01, and ^▲▲▲^ *p* < 0.001. ACTL6A, actin‐like protein 6A; ESCC, esophageal squamous cell carcinoma; RT, radiation therapy.

At 24, 48, 72, and 96 h after radiotherapy (RT), the CCK‐8 assay revealed that the ACTL6A knockdown group exhibited significantly diminished cell survival compared to the negative control (NC) group (*p* < 0.05) (Figure 2a). The experiment assessing colony development indicated that cells deficient in ACTL6A produced a markedly lower number of colonies compared to the negative control group (Figure 2b), indicating that ACTL6A knockdown significantly enhanced the radiosensitivity of ESCC cells. The wound healing assay demonstrated that ESCC cells with ACTL6A knockdown displayed a markedly diminished ability to close wounds compared to those in the negative control group (*p* < 0.05, Figure 2c). After ACTL6A knockdown, a significant decrease was observed in the invasive capability of KYSE150 and KYSE70 cells compared to the NC group (*p* < 0.05, Figure 2d). Western blot analysis was employed to validate the expression levels of key EMT markers, including E‐cadherin, N‐cadherin, and vimentin (Figure 2e). The shACTL6A group revealed higher E‐cadherin levels and lower vimentin and N‐cadherin expression in irradiated and non‐irradiated conditions (*p* < 0.05), implying that ACTL6A may facilitate EMT, thereby enhancing ESCC cell migration and invasion.

### 3.4 ACTL6A Knockdown Facilitates the Transition of ESCC Cells From the G0/G1 Phase to the G2/M Phase

**FIGURE 3:**
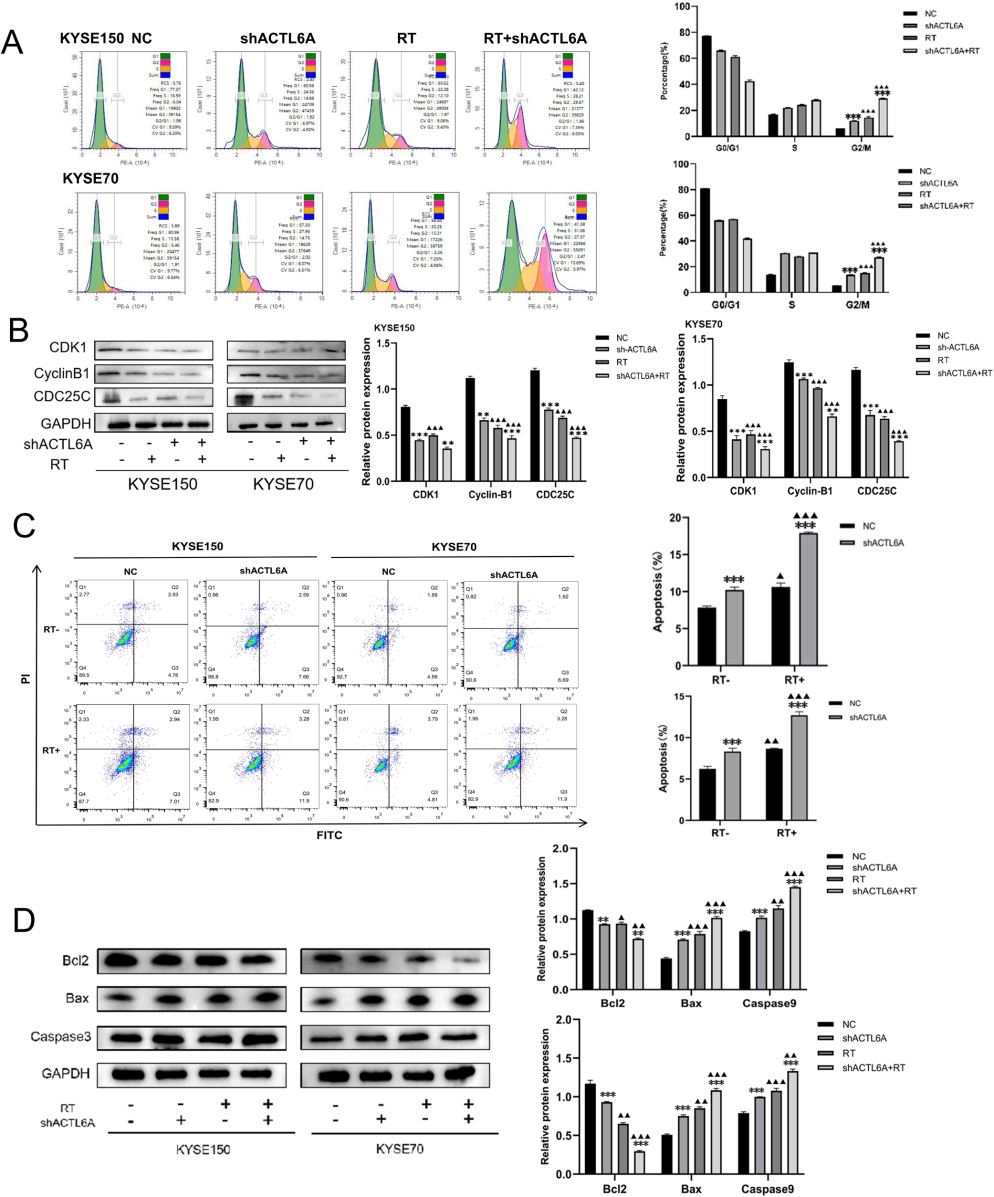
Regulation of cell cycle distribution and apoptosis in ESCC cells by ACTL6A. (a) Knockdown of ACTL6A alleviated G0/G1 phase block in KYSE150 and KYSE70 cells after radiotherapy. (b) ACTL6A affected the G0/G1 phase block of KYSE150 and KYSE70 cells after radiotherapy by regulating CDK1, CyclinB1, and CDC25 in the cells. (c) The apoptosis rate of cells in each group was detected by flow cytometry. (d) After IR, BCL2, BAX, and Caspase9 proteins were regulated by ACTL6A in KYSE150 and KYSE70 cells. Comparisons with the NC group are indicated by asterisks *p < 0.05, **p < 0.01, and ***p < 0.001; comparisons with the corresponding nonirradiated group are indicated by triangles ^▲^ *p* < 0.05, ^▲▲^ *p* < 0.01, and ^▲▲▲^ *p* < 0.001.

Flow cytometry (FCM) analysis of the cell cycle distribution revealed that G0/G1 phase arrest was alleviated in the ACTL6A knockdown group compared to the NC group in both ESCC cell lines (Figure 3a). After radiation exposure, G0/G1 phase arrest was significantly reduced in the ACTL6A knockdown group compared to the NC group (*p* < 0.05). The combination of ACTL6A knockdown with radiation resulted in a higher proportion of cells in the G2/M phase than in either ACTL6A knockdown or irradiation alone (*p* < 0.05), indicating that ACTL6A knockdown facilitates the transition of ESCC cells from the G0/G1 phase to the G2/M phase. The expression of essential cell cycle regulatory proteins, including CDK1, Cyclin B1, and CDC25C, was evaluated using Western blotting. The study demonstrated that CDK1, Cyclin B1, and CDC25C expression levels were downregulated in the ACTL6A knockdown group compared to the NC group, regardless of radiation exposure (Figure 3b), indicating that ACTL6A regulates cell cycle distribution in ESCC cells.

### 3.5 ACTL6A Knockdown Promotes Apoptosis in ESCC Cells

FCM revealed that the percentage of apoptotic cells in KYSE150 cells increased from 7.69% in the NC group to 10.25% in the ACTL6A knockdown group. After irradiation, the apoptotic cell percentages were 9.95% in the NC group and 17.8% in the ACTL6A knockdown group (Figure 3c). In KYSE70 cells, the apoptotic cell proportions were 6.44% in the NC group and 8.61% in the ACTL6A knockdown group. After irradiation, these proportions increased to 8.6% in the NC group and 12.4% in the ACTL6A knockdown group (Figure 3c). In addition, regardless of irradiation, Bax and caspase‐9 protein expression levels were higher in both ESCC cell lines. The ACTL6A knockdown group exhibited a significant reduction in Bcl‐2 protein expression (Figure 3d). These findings indicated that ACTL6A knockdown enhanced apoptosis in ESCC cells.

### 3.6 ACTL6A Knockdown Inhibits RT‐Induced DNA Damage Repair in ESCC Cells

**FIGURE 4:**
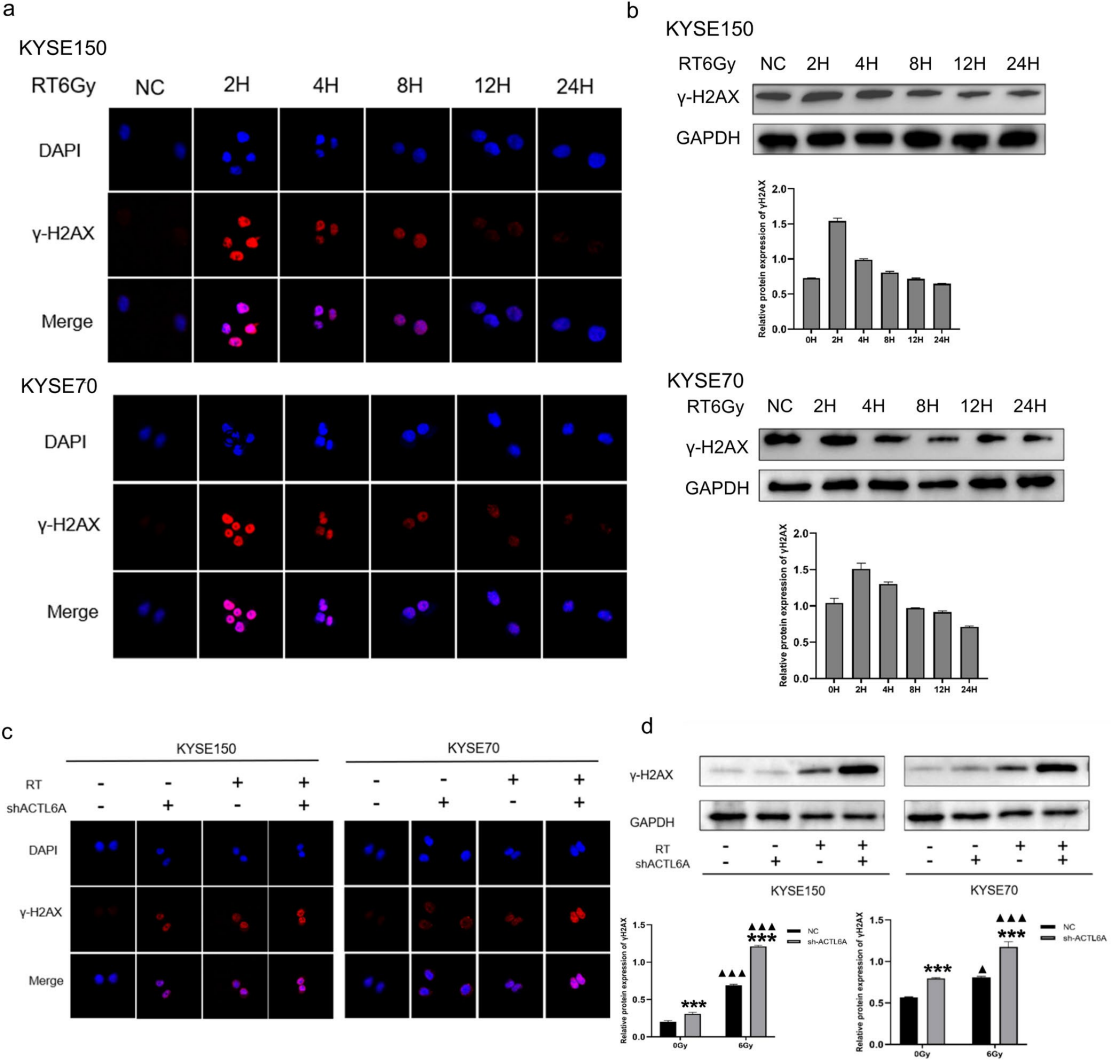
Participation of ACTL6A in DNA damage repair by regulating γH2AX protein expression. (a) Immunofluorescence analyses were used to observe the relationship between γH2AX protein expression and radiation exposure time in KYSE150 and KYSE70 cells. (b) The relationship between γH2AX expression level and radiation exposure time was analyzed by Western blot. (c) Following 6 Gy radiation exposure, immunofluorescence was used to observe how ACTL6A regulates γH2AX protein expression and participates in DNA damage repair. (d) The relationship between γH2AX expression level and ACTL6A was analyzed by Western blot. Statistical significance is indicated by asterisks: *p < 0.05, **p < 0.01, and ***p < 0.001 compared to the NC group; and by triangles: ^▲^ *p* < 0.05, ^▲▲^ *p* < 0.01, and ^▲▲▲^ *p* < 0.001 compared to the corresponding non‐irradiated group.

The DNA damage marker, γH2AX expression, was assessed in ESCC cells using immunofluorescence and Western blotting analysis. In KYSE150 and KYSE70 cell lines, γH2AX expression peaked at 2 h after irradiation, compared to other time intervals (0, 4, 8, 12, and 24 h) (Figure 4a,b). Furthermore, irradiated cells exhibited significantly higher γH2AX levels than non‐irradiated controls (Figure 4c). The findings of the immunofluorescence experiments were consistent with the trends observed in the Western blotting analysis (Figure 4d). The group that received combined treatment with knockdown exhibited higher γH2AX protein expression levels than the ESCC cells subjected solely to RT. This indicates that ACTL6A knockdown inhibits DNA repair, significantly increasing unrepaired DNA double‐strand breaks, thereby enhancing ESCC cell radiosensitivity.

### 3.7 ACTL6A Promotes EMT in ESCC Through Activation of the Wnt/β‐Catenin Signaling Pathway

**FIGURE 5:**
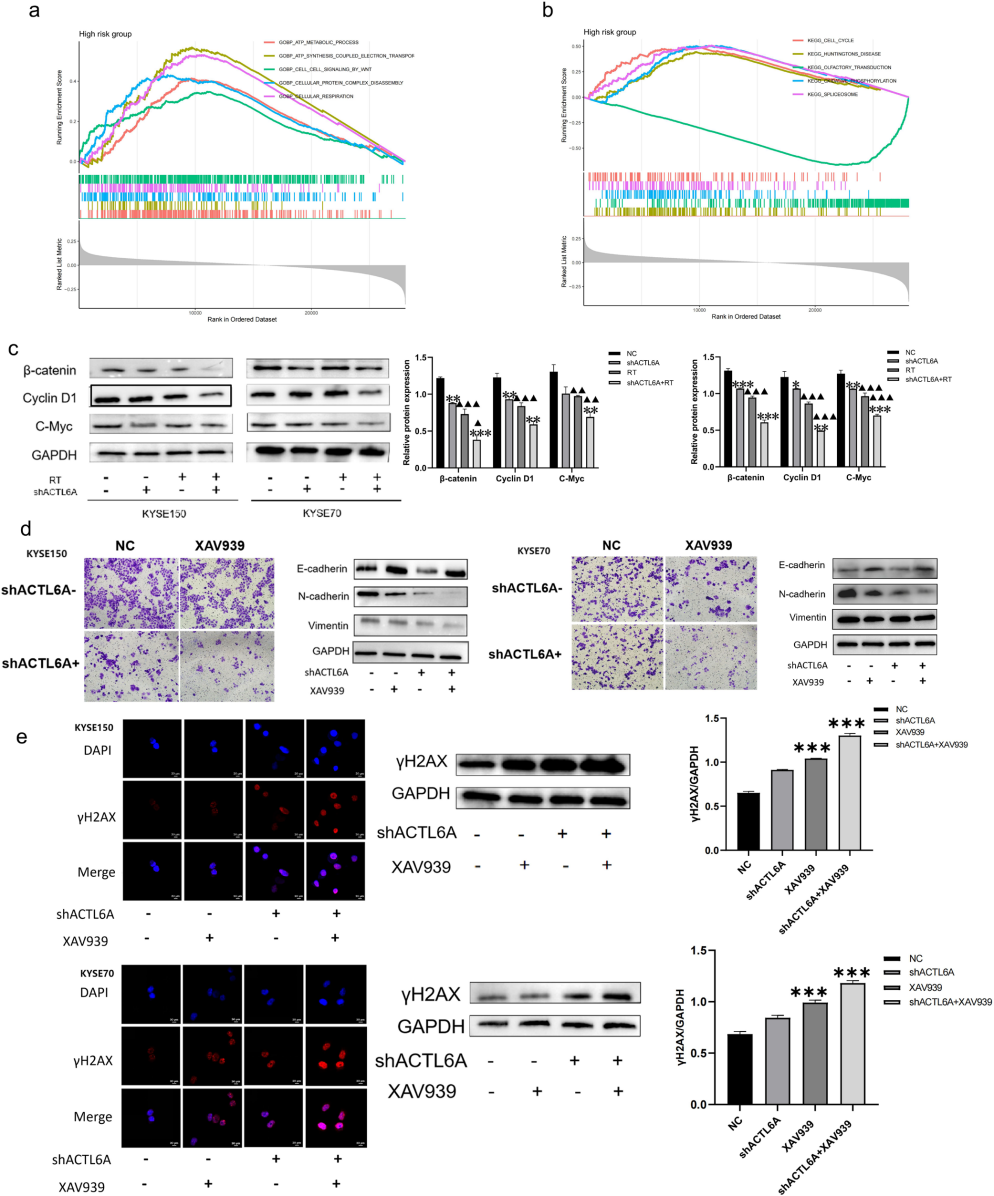
ACTL6A mediates EMT in ESCC Cells via the Wnt/β‐Catenin signaling pathway. (a) GO analysis of biological processes and KEGG pathways by GSEA. (b) Results of subcutaneously transplanted tumors in mice. (c) Western blot analysis of β‐catenin, Cyclin D1, and c‐Myc in KYSE150 and KYSE70 cell lines. (d) Wnt inhibitor XAV939 verified the effect of the knockdown of ACTL6A on the invasive ability and EMT‐related protein expression levels of ESCC cells. (e) Wnt inhibitor XAV939 verified the effect of the knockdown of ACTL6A on DNA damage repair in esophageal squamous carcinoma cells.

Gene set enrichment analysis (GSEA) of ACTL6A expression levels revealed that the ACTL6A high‐expression group significantly enriched the Wnt signaling pathway and other pathways linked to cancer (Figure 5a,b). Our previous studies evaluated EMT‐related marker expression in ACTL6A knockdown cells using Western blotting analysis. We performed additional Western blotting analysis to determine if ACTL6A affects EMT through the Wnt signaling pathway. The findings revealed that β‐catenin, cyclin D1, and c‐Myc expression levels were decreased by ACTL6A deletion (Figure 5c). Our effects of the Wnt inhibitor XAV939 on EMT reversion, DNA damage, and invasion ability. The results of the experiments showed that the Wnt pathway inhibitor, XAV939 (5 μM, 48 h) treatment significantly inhibited the invasive capacity of the esophageal squamous carcinoma cells. The number of perforated cells in the Wnt inhibitor XAV939 group compared with the NC group on the knockdown of ACTL6A was more pronounced in the shACTL6A + Wnt inhibitor XAV939 group than in the shACTL6A group alone. The difference was statistically significant (*p* < 0.05) and induced the EMT reversal, upregulation of E‐cadherin, and downregulation of N‐cadherin and vimentin, respectively. The difference was statistically significant (Figure 5d, *p* < 0.05). Meanwhile, the Wnt pathway inhibitor XAV939 led to an increase in the number of γH2AX foci of the DNA damage marker compared with the NC group. At ACTL6A knockdown, the number of γH2AX foci increased more in the shACTL6A + Wnt inhibitor XAV939 group than in the shACTL6A group alone (Figure 5e, *p* < 0.05). These phenotypes and molecular changes were highly consistent with the ACTL6A knockdown group, confirming that Wnt pathway inhibition synergistically regulated the invasive ability and radiosensitivity by reversing EMT and exacerbating DNA damage.

### 3.8 ACTL6A Knockdown Enhances Radiosensitivity in ESCC Xenograft Tumor Model

**FIGURE 6:**
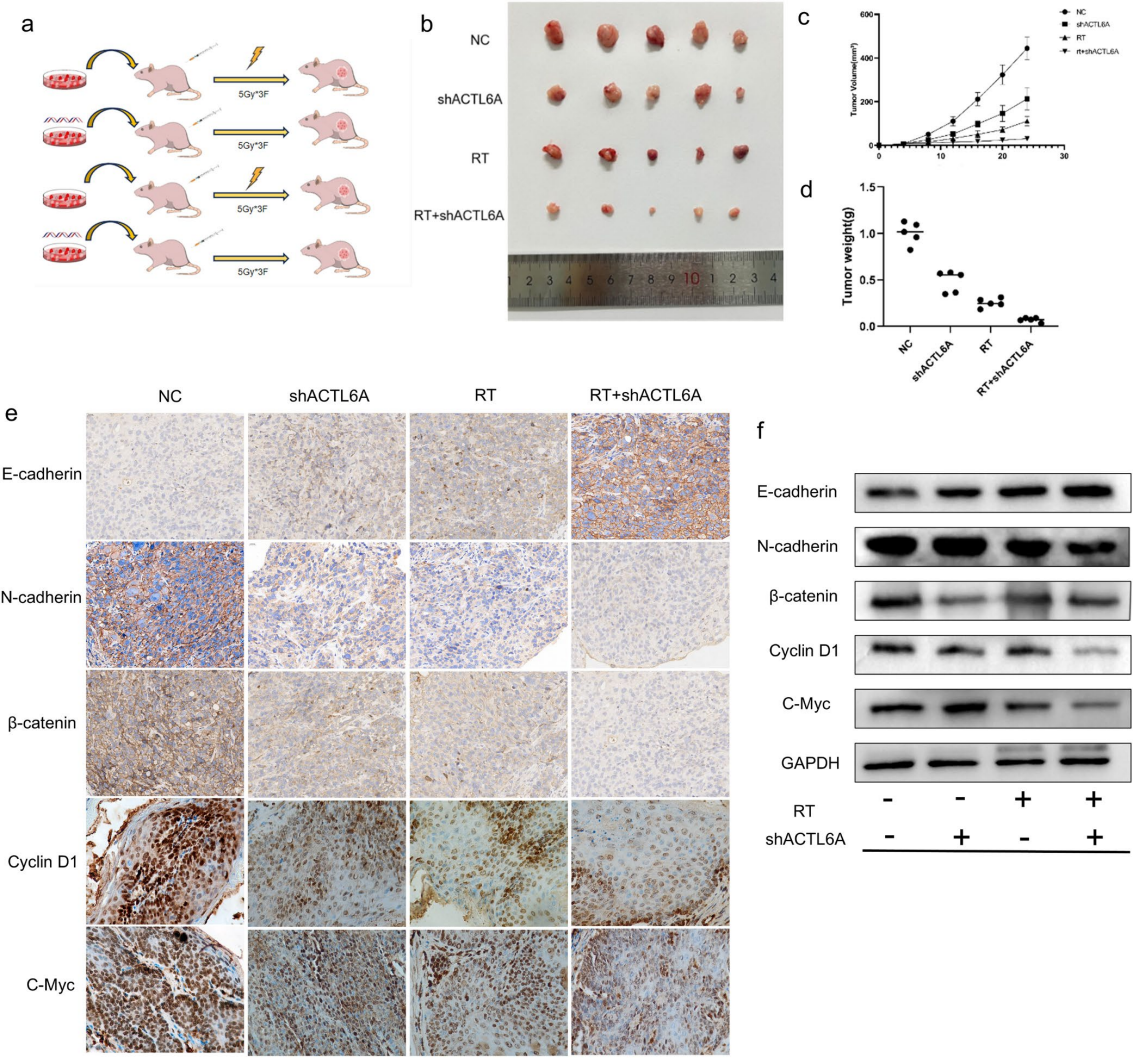
In vivo effects of ACTL6A on the radiation sensitivity of esophageal squamous cell carcinoma. (a) Schematic diagram of the animal experiment protocol. (b) Changes in tumor size: Images of tumors excised from BALB/c nude mice after receiving radiation and non‐radiation treatments. (c) Tumor volume changes: Statistical curves showing the effect of ACTL6A knockdown with or without radiation on tumor volume. (d) Tumor weight changes: Final tumor weights following ACTL6A knockdown and radiation or non‐radiation treatments. (e) Immunohistochemical and (f) Western Blot Analysis of E‐cadherin, N‐cadherin, β‐catenin, Cyclin D1, and c‐Myc expression. Original magnification, ×400.

An ESCC xenograft tumor model was successfully created by subcutaneously injecting KYSE150‐NC and KYSE150‐shACTL6A cells into the BALB/c nude mice. They were treated solely with shACTL6A, RT alone, and RT combined with shACTL6A (Figure 6a). Regardless of radiotherapy, results demonstrated that tumor sizes measured biweekly were consistently smaller in the shACTL6A group than in the NC group. Furthermore, the tumors were bigger in the blank control, shACTL6A alone, and RT alone groups than in the RT coupled with shACTL6A group (Figure 6b). The subcutaneous tumors in the RT coupled with the shACTL6A group almost stopped developing on Day 15 (Figure 6c), indicating that tumor progression was suppressed in the shACTL6A and RT combined with shACTL6A groups. The tumor weight was significantly reduced in the shACTL6A and RT combined with shACTL6A groups compared to the control and RT‐only groups (Figure 6d). The tumor tissues were obtained from each mouse group, and the protein levels of EMT and WNT pathway‐related markers (including E‐cadherin, N‐cadherin, β‐catenin, cyclin D1, and c‐Myc) were assessed (Figure 6e). The results revealed that E‐cadherin levels in the subcutaneous xenografts of mice in the shACTL6A group exhibited a significant decrease compared to those in the control group, regardless of RT, while N‐cadherin levels exhibited an increase. The expression levels of β‐catenin, Cyclin D1, and c‐Myc were consistently lower in the shACTL6A group than in the control group, irrespective of the radiation intervention (Figure 6f). After homogenizing tumor tissues and extracting proteins, Western blotting analysis confirmed the immunohistochemistry findings.

## 4 Discussion

**FIGURE 7:**
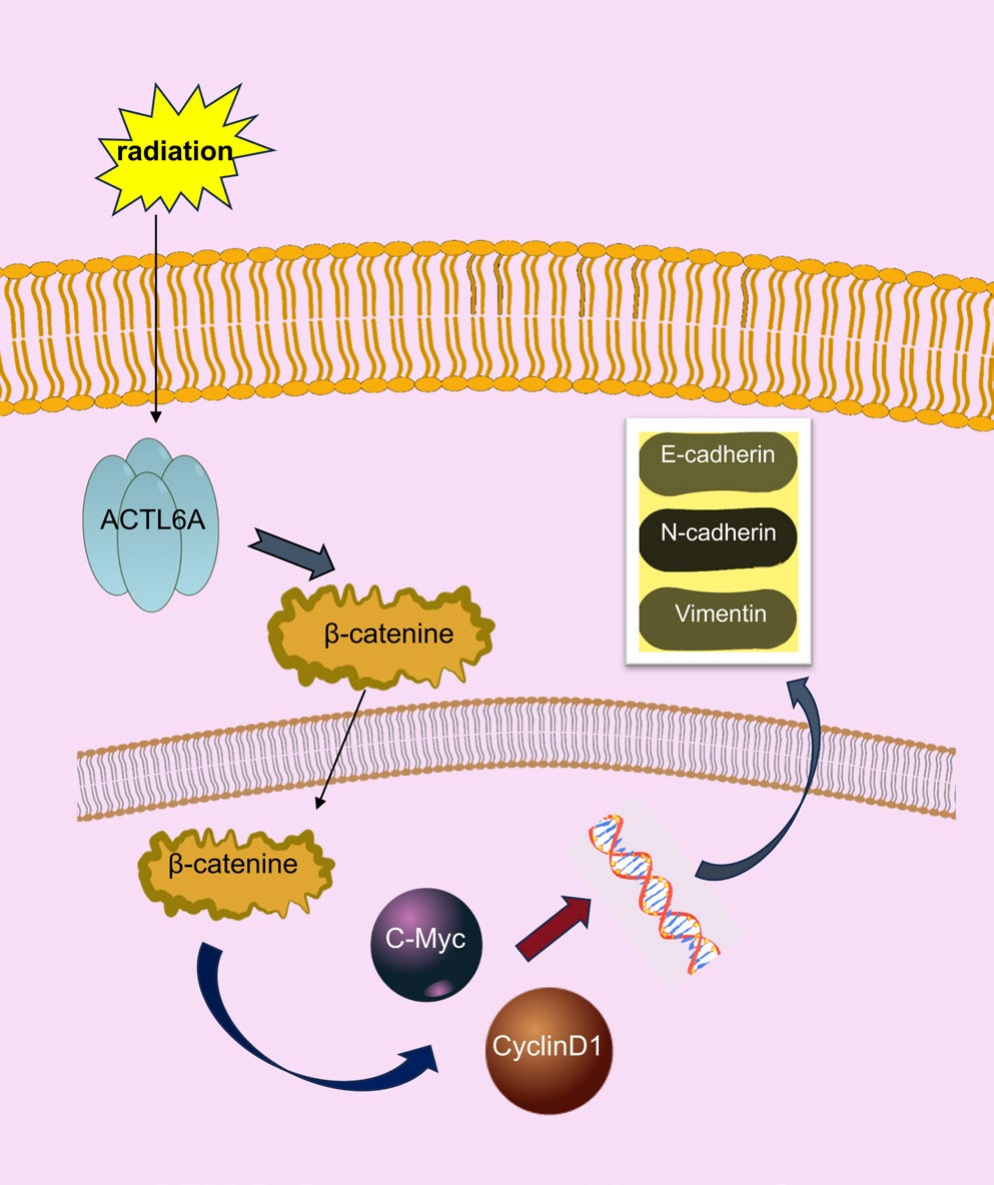
Schematic diagram of the effects of ACTL6A on radiotherapy sensitivity in ESCC.

ESCC, a highly aggressive malignant tumor, is commonly diagnosed at a stage of metastasis or localized progression [3]. Radiotherapy is an essential treatment modality for ESCC, especially when surgical resection is unfeasible. Clinical resistance of tumor cells to radiotherapy constitutes a significant impediment to treatment efficacy. Despite high radiation doses, achieving complete tumor control and radiation resistance may result in recurrence and metastasis [18]. Consequently, overcoming radiotherapy tolerance and enhancing radiosensitivity are crucial for improving prognosis and extending survival time in patients with ESCC. This research explored the function and underlying mechanisms of ACTL6A in modulating the radiosensitivity of ESCC cells. The findings indicated that ACTL6A was highly expressed in ESCC cells, and its knockdown enhanced the radiosensitivity of these cells, implying that ACTL6A may be a significant therapeutic target for ESCC (Figure 7).

ACTL6A is an essential accessory subunit of the SWI/SNF chromatin remodeling complex and is found in all isoforms. Prior investigations indicate that ACTL6A is involved in several cancer types. The abnormal expression of the *ACTL6A* gene can function as a transcription factor, modulating the expression of essential differentiation‐related genes, thereby affecting the differentiation and proliferation of tumor cells and their migration and invasion through the regulation of EMT‐related genes [19, 20]. Besides, abnormal expression of the *ACTL6A* gene can impact the functionality of the SWI/SNF chromatin remodeling complex, thereby affecting the DNA repair capabilities of tumor cells and facilitating the onset and progression of malignant behavior [21]. This study reveals that *ACTL6A* gene expression in ESCC is significantly higher, and its high expression correlates with low patient survival time. The significantly increased expression level of ACTL6A protein in ESCC indicates its potential role in facilitating the malignant behavior of ESCC cells. Analysis of the expression level of ACTL6A in tumor tissues of ESCC patients revealed that patients with high ACTL6A expression had worse OS and DFS than those with low ACTL6A expression. This suggests that patients with high ACTL6A expression have a poorer prognosis, and ACTL6A may be a new therapeutic target for ESCC patients. Further analysis of the clinical characteristics of patients in the clinical dataset found that the expression of ACTL6A was related to T stage and TNM stage, which may also be the reason why ESCC patients with high ACTL6A expression have a poorer prognosis.

ACTL6A protein expression levels varied among several ESCC cell lines, with higher levels observed in KYSE150 and KYSE70 cell lines than in other cell lines. CCK8 assays revealed a reduction in *ACTL6A* gene expression, which may impede the proliferation of ESCC cells, regardless of radiotherapy intervention. The plate colony experiment revealed that ESCC cells with knocked‐down *ACTL6A* gene expression exhibited significantly higher sensitivity to radiotherapy than the control group, indicating a strong correlation between ACTL6A and the radiosensitivity of ESCC cells. The above results are consistent with those of previous findings in other types of tumors, where the *ACTL6A* gene was identified as a promoter of epithelial squamous cell carcinoma [22]. Similarly, the *ACTL6A* gene can facilitate the malignant progression of glioma by promoting the proliferation of glioma cells [23]. The aberrant expression of ACTL6A in rhabdomyosarcoma maintains tumor cells in an undifferentiated state and enhances their robust proliferation and differentiation capacity through modulation of SWISNF chromatin remodeling complex function [24].

Previous studies reported that high expression of the *ACTL6A* gene enhances the invasiveness of various tumor cells in breast cancer, glioma, and colorectal cancer. The *ACTL6A* gene is involved in signaling pathway regulation related to epithelial‐mesenchymal transition [25, 26, 27]. The invasion and migration ability of ESCC cells was assessed through in vitro experiments. The above findings indicate that the combined radiotherapy treatment led to a lower invasion and migration ability of ACTL6A knockdown cells in the ACTL6A knockdown group than in the control group, implying that ACTL6A plays a promoting role in the invasion and migration of ESCC cells. Furthermore, ACTL6A may affect the radiosensitivity of these cells by impacting their invasion and migration capabilities. This study demonstrated a significant increase in apoptosis levels in the ACTL6A knockdown group, and the cell cycle migrated from the G1/G0 phase to the G2/M phase. Previous studies reported that ACTL6A in head and neck squamous cell carcinoma (HNSCC) cells can be co‐amplified with p63 to maintain tumor cells in their undifferentiated state and promote their regenerative proliferation. Additionally, *ACTL6A* gene knockdown in HNSCC can inhibit the cell cycle at the G1/G0 phase [28]. These findings suggest that ACTL6A regulates apoptosis, cell cycle, and ESCC cell progression, attenuating ESCC cell sensitivity by inhibiting apoptosis and impeding the transition from the G1/G0 to the G2/M phase.

Ionizing radiation can induce DNA damage in cells, primarily by causing DNA double‐strand breaks. DNA damage includes base modifications, intrastrand and interstrand cross‐links, single‐strand breaks, and double‐strand breaks (DSB). Among these, DSBs are considered the most harmful DNA damage. If not repaired promptly, it will compromise the integrity and stability of the genome, resulting in cell cycle arrest or cell death [29]. A previous study reported that when cells undergo DNA damage, especially DSBs, the serine residue at position 139 on the C‐terminus of H2AX is phosphorylated into γH2AX [30]. Gamma‐H2AX is the starting signal molecule for DNA DSBs, facilitating the recruitment of various DNA damage response proteins to the site of DNA DSBs and forming a DNA damage response functional complex that activates and initiates the DNA repair mechanism. After DNA repair is completed, γH2AX undergoes a rapid dephosphorylation process. Consequently, γH2AX is a sensitive marker of DNA damage or restoration. Herein, the expression level of γH2AX protein in ESCC cells of the ACTL6A knockdown group is significantly increased after radiotherapy. Hence, it is hypothesized that ACTL6A may inhibit the radiation‐induced DNA damage repair in ESCC cells by suppressing their radiosensitivity. Consistent with this, a recent study in non‐small cell lung cancer also demonstrated that ACTL6A depletion enhances radiation‐induced γH2AX accumulation by impairing DNA DSB repair efficiency, further supporting the conserved role of ACTL6A in regulating DNA damage repair across different tumor types [31]. Additionally, the expression of ACTL6A exhibited a strong association with the cell cycle of ESCC and the Wnt/β‐catenin signaling pathway. Western blotting revealed that ACTL6A knockdown suppressed the activity of this signaling pathway. The Wnt/β‐catenin signaling pathway is a classical EMT‐related pathway associated with tumor invasion and metastasis [32], corroborating earlier findings within the context of non‐small cell lung cancer, which demonstrated that activation of this pathway facilitates EMT [33]. Notably, a separate study focusing on hepatocellular carcinoma identified ACTL6A as a key activator of the Wnt/β‐catenin pathway, where its overexpression promoted nuclear translocation of β‐catenin and upregulation of downstream EMT‐related genes, a mechanism that aligns with our observations in ESCC [34]. We used the effect of Wnt inhibitor XAV939 on EMT reversal, DNA damage, and invasion ability, and found that Wnt inhibitor XAV939 and knockdown of ACTL6A inhibited the invasion ability and DNA damage repair, indicating that knockdown of ACTL6A may inhibit EMT and promote γH2AX through Wnt cell pathway, which then promoted the radiosensitivity of ESCC cells.

The in vitro experimental results are consistent with those from in vivo experiments. The xenograft tumor size was significantly reduced in the ACTL6A knockdown group compared to the control group. In the combined radiotherapy, the xenograft tumor volume was significantly reduced in the knockdown group + RT group compared to the RT group. These findings indicate that ACTL6A knockdown enhances ESCC radiosensitivity by inhibiting ESCC cell proliferation. The in vivo findings in mice are consistent with those of our previous cellular experiments. Accordingly, we hypothesized that ACTL6A facilitates EMT in ESCC by activating the Wnt/β‐catenin signaling pathway.

It can be hypothesized that ACTL6A expression promotes ESCC proliferation, promotes ESCC progression, and inhibits ESCC sensitivity to radiotherapy. This function may be realized by altering invasion, migration, apoptosis, cell cycle, and inhibiting DNA damage repair in ESCC, potentially by promoting the activity of the Wnt/β‐catenin signaling pathway.

In conclusion, our study demonstrated that the knockdown of ACTL6A enhanced the radiosensitivity of esophageal squamous cell carcinoma. This was achieved by inhibiting the Wnt/β‐Catenin signaling pathway, which in turn led to the suppression of EMT. These findings provide novel insights into the molecular mechanisms underlying esophageal squamous cell carcinoma radiosensitivity and may offer potential therapeutic targets for this disease.

## Author Contributions

**Shuo Zhou** and **Tongyou Sun:** conceptualization, methodology, software, investigation, formal analysis, data curation, writing – original draft. **Likun Liu** and **Dong Guo:** investigation. **Xueyuan Zhang:** visualization, investigation. **Wenbin Shen:** resources, supervision. **Shuchai Zhu** and **Shaolin Gao:** funding acquisition, resources, supervision, writing – review and editing. All authors read and approved the final manuscript.

## Disclosure

The authors have nothing to report.

## Conflicts of Interest

The authors declare no conflicts of interest.

## Data Availability

The data supporting the findings of this study are included within the article. Additional inquiries can be directed to the corresponding author.
